# Mediating role of triglyceride glucose-related index in the associations of composite dietary antioxidant index with cardiovascular disease and mortality in older adults with hypertension: a national cohort study

**DOI:** 10.3389/fnut.2025.1574876

**Published:** 2025-04-23

**Authors:** Yajie Zhang, Yingying Liu, Huiquan Qiao, Qiongqiong Ma, Bing Zhao, Qian Wu, Hongyu Li

**Affiliations:** ^1^Department of Obstetrics and Gynecology, The Third Affiliated Hospital of Zhengzhou University, Zhengzhou, China; ^2^Department of Biomedical Sciences, Institute for Medical Science, Jeonbuk National University Medical School, Jeonju, Republic of Korea

**Keywords:** composite dietary antioxidant index, cardiovascular disease, triglyceride glucose-related index, mortality, national health and nutrition, hypertension

## Abstract

**Background:**

This research investigates the relationships between the composite dietary antioxidant index (CDAI) and the likelihood of cardiovascular disease (CVD) and mortality in older adults with hypertension. Utilized data from the National Health and Nutrition Examination Survey (NHANES) to investigate the potential mediating role of the triglyceride-glucose (TyG) index in these relationships.

**Methods:**

A cohort of 5,276 participants, aged 65 years or older and diagnosed with hypertension, was extracted from the NHANES database. The main outcomes examined were the odds of CVD and mortality, utilizing data from the National Center for Health Statistics (NCHS). Multivariate logistic regression models were utilized to evaluate the relationship between CDAI and CVD. Cox proportional hazards regression models and Kaplan–Meier survival curves were utilized to analyze the relationship between CDAI and mortality. Mediation analysis was conducted to assess the potential intermediary role of TyG-related indicators—specifically TyG, TyG-BMI, TyG-WC, and TyG-WHtR— in the connection between CDAI and mortality.

**Results:**

The mean CDAI for the study participants was 1.88 ± 3.90, and the average age was 74.15 ± 5.96 years. During an average follow-up duration of 109.51 months, 4,712 cases of CVD and 725 recorded deaths were observed. In the fully adjusted models, CDAI showed a negative association with both CVD (Odds Ratio [OR] = 0.94, 95% Confidence Interval [CI] = 0.92–0.97) and mortality (Hazard Ratio [HR] = 0.95, 95% CI = 0.93–0.97). Mediation analysis indicated that the TyG-BMI, TyG-WC, and TyG-WHtR indices accounted for 33.1%, 34.3%, and 19.1% of the relationship between CDAI and mortality, respectively.

**Conclusion:**

A higher CDAI demonstrated an inverse association with both CVD and mortality in elderly hypertensive individuals. The relationship was partially mediated by TyG-related indices, indicating that increased antioxidant intake may lead to improved health outcomes and a decreased risk of poor prognosis in this population.

## 1 Introduction

The inaugural WHO Global Hypertension Report highlights hypertension as a significant global health concern, impacting 140 million individuals worldwide and substantially contributing to CVD and overall mortality, particularly among those aged 65 years and older (1). Hypertension, defined as persistently elevated blood pressure, is a well-established risk factor for cardiovascular diseases (CVD), including heart failure, chronic kidney disease, and stroke (2, 3). It contributes to the development and progression of these conditions by inducing structural and functional changes in the heart and blood vessels, such as left ventricular hypertrophy, arterial stiffness, and endothelial dysfunction (4, 5). In the United States, the prevalence of CVD in individuals over the age of 60 has surpassed 78% (6), with healthcare costs exceeding $89.3 billion (7), Consequently, CVD emerges as the primary determinant affecting life expectancy in the population aged over 75 years (8). As the global population ages, the prevalence of hypertension is expected to increase, emphasizing the urgent need for a comprehensive understanding of its determinants to inform the development of effective prevention and management strategies.

Oxidative stress is a critical pathophysiological mechanism that facilitates the development and advancement of hypertension (9). Dietary components, particularly those rich in antioxidants, are implicated in hypertension development (10, 11). The CDAI, a measure of dietary antioxidant levels, is associated with multiple clinical disorders, including cancer, metabolic syndrome, cardiovascular illnesses, and diabetes (12–14). Antioxidants, such as vitamins C and E, along with carotenoids, maintain endothelial function by neutralizing free radicals and reducing oxidative stress, thereby potentially inhibiting the onset and progression of hypertension (15–17). Beyond their direct antioxidant effects, dietary antioxidants may also improve insulin sensitivity by mitigating oxidative stress and inflammation, which are key contributors to insulin resistance (IR) (18, 19). The TyG index, a surrogate marker of IR, has been shown to predict the incidence of diabetes, hypertension, and CVD (20–24). Mechanistically, antioxidants may reduce IR by enhancing insulin signaling pathways and improving glucose metabolism, thereby indirectly benefiting cardiovascular health (25).

In this study, we hypothesize that a higher CDAI is associated with a reduced risk of CVD and mortality among elderly hypertensive patients, and that this association is partially mediated by TyG-related indices, reflecting the role of insulin resistance in linking antioxidant intake to cardiovascular outcomes. Based on a nationally representative sample, we aim to address the following research questions: (1) What is the association between CDAI and the odds of CVD occurrence in elderly hypertensive patients? (2) Does CDAI predict mortality in this population? (3) To what extent do TyG-related indices mediate the relationship between CDAI and cardiovascular outcomes? By elucidating these relationships, this study aims to provide novel insights into the role of dietary antioxidants in hypertension management and CVD prevention.

## 2 Materials and methods

### 2.1 Data source

This study utilized data from the NHANES, managed by the National Center for Health Statistics (NCHS), an entity within the Centers for Disease Control and Prevention (CDC). NHANES is primarily designed to evaluate the health and nutritional status of the US population. The survey’s protocols, approved by the NCHS Institutional Review Board, ensure compliance with ethical guidelines, including the acquisition of informed consent from all participants. Each participant provided written consent, signifying their understanding and agreement to participate. The dataset used in this study is accessible to the public on the NHANES website at https://www.cdc.gov/nchs/nhanes/index.html.

### 2.2 Study population

**FIGURE 1:**
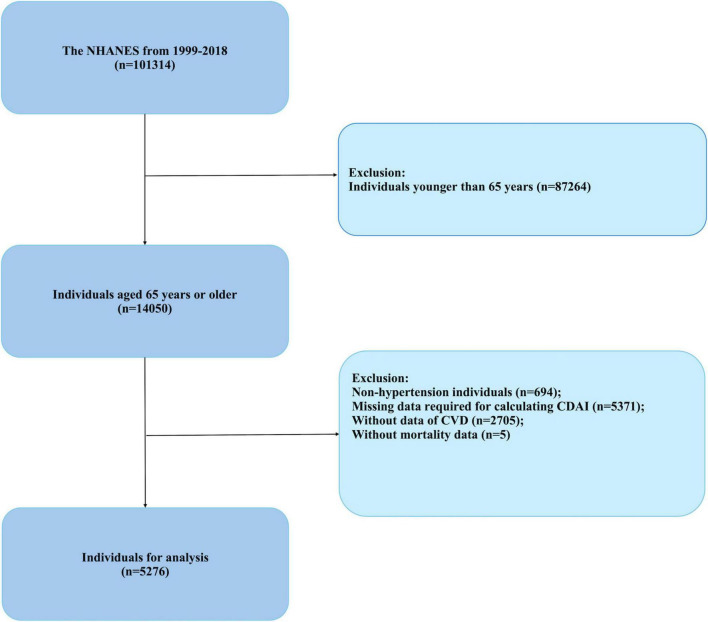
Flowchart of study participants (NHANES, 1999–2018).

Trained medical professionals conducted blood pressure assessments using a mercury sphygmomanometer at mobile examination centers. Participants were seated, with the right arm typically employed for measurements unless there were compelling reasons to use the left. Following a 5-min rest period, blood pressure was recorded on three occasions, and the mean of these measurements was utilized in subsequent analyses. Hypertension was defined using one or more of the following criteria: a systolic blood pressure of 140 mmHg or higher, a diastolic blood pressure of 90 mmHg or higher, a self-reported diagnosis of hypertension by a healthcare provider, or the use of antihypertensive medications (26). Participants were excluded from the study if they were under the age of 65, did not have hypertension, or had incomplete data regarding the CDAI, cardiovascular disease, or survival data. The final cohort for analysis comprised 5,276 individuals, with data extracted from the NHANES conducted between 1999 and 2018 (Figure 1).

### 2.3 Assessment of CDAI

Within the NHANES protocol, dietary assessments were conducted by qualified personnel who administered two 24-h dietary recall interviews to participants. The first interview took place in-person, while the second was conducted via telephone within a 3 to 10-day interval following the initial encounter. The average antioxidant intake derived from the two dietary recall periods was employed for statistical analysis to reduce potential bias. The CDAI levels were determined for each participant using an adapted version of the method established by Wright et al. (27), which incorporates the dietary intake of six antioxidants: vitamins A, C, E, carotenoids, zinc, and selenium. Each nutrient’s intake was normalized by adjusting for the population mean and standard deviation, and these normalized values were then summed to determine the CDAI, as further elaborated in a referenced study.


$$\text{CDAI}=\sum_{\text{i}=1}^{\text{n}=6}\frac{\text{individual}\,\text{Intake}-\mathrm{Mean}}{\text{SD}}$$

### 2.4 Assessment of CVD

CVD diagnoses were determined by self-reported confirmations from physicians, collected during structured interviews using a standardized medical questionnaire. Specifically, participants were asked, “Have you ever been diagnosed by a healthcare professional with congestive heart failure, coronary heart disease, angina pectoris, myocardial infarction, or stroke?” Positive answers to any of these symptoms were criteria for diagnosing an individual with CVD. Definitions for congestive heart failure, myocardial infarction, angina pectoris, and coronary heart disease were consistent with established medical criteria for these conditions (28).

### 2.5 Survival status

Mortality ascertainment was facilitated by leveraging the NHANES public use linked mortality file, which was current as of December 31, 2019, alongside the National Death Index (NDI) from the National Center for Health Statistics (NCHS). A probabilistic matching algorithm was employed to link these datasets. The International Statistical Classification of Diseases and Related Health Problems, 10th Revision (ICD-10), served as the framework for identifying the underlying causes of death. Specifically, cardiovascular mortality was categorized based on ICD-10 codes for heart diseases (I00–I09, I11, I13, I20–I51) and cerebrovascular diseases (I60–I69) (29).

### 2.6 Covariates

Based on the previous literature, variables associated with hypertension and dietary habits were incorporated. Sociodemographic factors were evaluated, encompassing aspects like age, sex, race, education attainment, relationship status, and the household’s poverty-to-income ratio (PIR). Anthropometric and lifestyle factors were also recorded, comprising body mass index (BMI), smoking history, evaluated using the inquiry, “Have you smoked at least 100 cigarettes in your lifetime?” [SMQ020], classified as Yes or No, and alcohol consumption (assessed using the query “Had at least 12 alcoholic drinks in the past year?” [ALQ101], categorized as Yes, No, or uncertain). The Diabetes Questionnaire (DIQ010) was utilized to ascertain diabetes status, inquiring if participants had ever received a diabetes diagnosis from a healthcare provider, excluding gestational diabetes. This resulted in three classifications: Yes, No, and Borderline.

Anthropometric measurements included waist circumference (WC) and waist-to-height ratio (WHtR). Biochemical profiling encompassed lipid panel analysis, including triglycerides (TG), total cholesterol (TC), high-density lipoprotein cholesterol (HDL-C), and low-density lipoprotein cholesterol (LDL-C). Hematological parameters, specifically neutrophil count, lymphocyte count, and platelet count, were utilized to compute the systemic immunoinflammatory index (SII), a novel marker of inflammation (30). Nutritional assessment focused on quantifying energy intake, protein consumption, and total fat intake.

Dietary quality was evaluated using two validated indices: the Dietary Inflammatory Index (DII) and the Healthy Eating Index (HEI). The DII, a previously validated tool comprising 45 dietary components (macronutrients, micronutrients, and flavonoids), was calculated based on 27 available components from NHANES 24-h dietary recall data (31). The HEI, a 0–100 scale metric developed collaboratively by the US Department of Agriculture and the National Cancer Institute, assesses adherence to the Dietary Guidelines for Americans through 13 components representing major food groups and essential nutrients, each scored between 5 and 10 points (32). Higher HEI scores indicate stronger alignment with recommended dietary patterns.

The formulas for calculating the triglyceride glucose-related indexes are as follows:

(1) $\text{TyG}=\text{ln}\,\left[\frac{\mathrm{triglycerides}\,\left(\mathrm{mg}/\mathrm{dl}\right)\times \mathrm{glucose}\,\left(\mathrm{mg}/\mathrm{dl}\right)}{2}\right]$;
(2) TyG−BMI = TyG × BMI, BMI = body mass (kg)/height (m^2^);
(3) TyG−WC = TyG × WC, WC = waistcircumference (cm);
(4) TyG−WHtR = TyG × WHtR, WHtR = waist circumference (cm)/height(cm);
(5) $S\,I\,I=\frac{\mathrm{Neutrophil}\,{\mathrm{count}}^{*}\,\mathrm{lymphocyte}\,\mathrm{count}}{\mathrm{platelet}\,\mathrm{count}}$.

### 2.7 Statistical analysis

Statistical analyses were performed following the guidelines established by the CDC.^1^ Participants were categorized into tertiles based on the CDAI scores: Low (−6.52 to −0.20), Middle (−0.20 to 2.75), and High (2.75 to 19.97). Continuous variables were expressed as weighted means accompanied by standard errors, whereas categorical variables were reported as unweighted frequencies along with weighted proportions. Group differences were evaluated through one-way analyses of variance (ANOVA) for continuous variables and chi-square tests for categorical variables. Missing data were handled using multiple imputation by chained equations (MICE) for continuous variables, while missing categorical variables were treated as a separate category to retain participants with incomplete data. To assess the statistical power of our findings, we conducted post-hoc sample size analysis, which confirmed that the study had sufficient power to detect significant associations between CDAI and the outcomes of interest. This analysis further strengthens the robustness of our results and supports the validity of the observed associations.

Weighted multivariate logistic regression analyses were employed to investigate the relationship between the CDAI and the prevalence of CVD, with results revealed as ratios of odds (ORs) and 95% confidence intervals (CIs) across three models. Three models were developed. Model 1 was a crude model with no covariate adjustment, Model 2 was adjusted for gender, age, race; and education level, and Model 3 included further adjustments for marital status, PIR, BMI, smoking, alcohol consumption, diabetes, caloric intake, total fat intake, protein intake, HDL-c, LDL-c, glucose, total cholesterol, triglycerides, DII, HEI2020 and SII.

Multivariate Cox proportional hazard models were utilized to estimate the associations of CDAI with mortality, with findings presented as hazard ratios (HRs) and 95% CIs. The identical three multivariate regression models originally outlined in this study were constructed. Kaplan–Meier curves were generated to display mortality risk by CDAI categories. We examine subgroup studies to identify potential differences among various populations, including age, gender, race, educational level, marital status, PIR, BMI, diabetes, smoking, and drinking subgroups.

Consistent with our previous approach to mediation studies (33), the mediation was confirmed if the indirect effect was significant, the total effect was significant, and the proportion mediated by the mediator was positive, after adjusting for all covariates as the Model 3. Mediation analysis was conducted using the “mediation” package in R (version 4.2.2) to assess the mediating effects of TyG-related indices (TyG, TyG-BMI, TyG-WC, and TyG-WHtR) as well as by inflammation-related indices (SII, neutrophils, and lymphocytes) on the relationship between CDAI and mortality (34). All statistical analyses were conducted using R statistical software (version 4.3.1; R Foundation for Statistical Computing, Vienna, Austria) and EmpowerStats software (version 4.2).

## 3 Results

### 3.1 Baseline characteristics

This study encompassed 5,276 participants (weighted 152,643,880) aged 65 years or older from NHANES spanning 1999–2018. As shown in Table 1, the mean age across all groups was 74.15 years. Higher CDAI scores were associated with younger age, male predominance, higher socioeconomic status, and lower systemic inflammation (*P* < 0.001). Participants in the high CDAI tertile exhibited lower fasting glucose (107.30 ± 22.36 mg/dL) and total cholesterol (192.98 ± 40.10 mg/dL) levels compared to the low tertile (*P* < 0.001). Additionally, the high CDAI group had reduced dietary inflammation (DII: 1.20 ± 1.58) and higher CVD prevalence (91.67%). However, smoking status, diabetes, HDL-C, LDL-C, energy intake, protein intake, and total fat intake did not show significant differences across the CDAI groups.

**TABLE 1:** Basic characteristics of participants (NHANES, 1999–2018).

^a^Characteristics	Total	CDAI			
		**Low (−6.52 to −0.20)**	**Middle (−0.20 to 2.75)**	**High (2.75–19.97)**	***p*-value**
***N***	5,276	1,767	1,766	1,743	
**Age (years)**	74.15 ± 5.96	74.62 ± 6.00	74.10 ± 5.93	73.74 ± 5.93	0.007
**Gender**					< 0.001
Male	2,214 (33.07%)	561 (23.30%)	787 (34.30%)	866 (41.60%)	
Female	3,062 (66.93%)	1,206 (76.70%)	979 (65.70%)	877 (58.40%)	
**Race/ethnicity, *n* (%)**					0.001
Maxican-American	698 (3.60%)	262 (4.16%)	222 (3.25%)	214 (3.38%)	
Other Hispanic	413 (4.14%)	137 (4.23%)	133 (3.69%)	143 (4.50%)	
Non-Hispanic White	2,970 (78.93%)	971 (77.86%)	1,013 (79.86%)	986 (79.08%)	
Non-Hispanic Black	798 (7.39%)	233 (6.55%)	275 (7.63%)	290 (8.00%)	
Other races	397 (5.94%)	164 (7.19 %)	123 (5.58%)	110 (5.04%)	
**Education level, *n* (%)**					< 0.001
< high school	4,384 (83.30%)	1,559 (89.09%)	1,454 (83.44%)	1,371 (77.40%)	
≥ high school	892 (16.70%)	208 (10.91%)	312 (16.56%)	372 (22.60%)	
**Marital status, *n* (%)**					< 0.001
Having a partner	2,759 (54.76%)	815 (49.42%)	951 (55.60%)	993 (59.24%)	
Not having a partner	2,517 (45.24%)	952 (50.58%)	815 (44.40%)	750 (40.76%)	
**Family PIR**	2.69 ± 1.38	2.61 ± 1.36	2.72 ± 1.37	2.74 ± 1.40	0.009
**BMI**	25.85 ± 3.63	24.56 ± 3.49	25.93 ± 3.41	27.05 ± 3.60	< 0.001
**Drinking alcohol, *n* (%)**					< 0.001
Yes	2,623 (50.04%)	780 (45.60%)	909 (51.53%)	934 (53.00%)	
No	1,795 (33.22%)	693 (37.77%)	563 (31.61%)	539 (30.29%)	
Unclear	858 (16.73%)	294 (16.63%)	294 (16.86%)	270 (16.71%)	
**Smoking, *n* (%)**					0.484
Yes	2,185 (41.63%)	750 (42.25%)	730 (41.33%)	705 (41.30%)	
No	3,091 (58.37%)	1,017 (57.75%)	1,036 (58.67%)	1,038 (58.70%)	
**Diabetes, *n* (%)**					0.582
Yes	581 (12.21%)	179 (11.21%)	208 (13.68%)	194 (11.77%)	
No	4,609 (86.20%)	1,557 (86.73%)	1,532 (85.10%)	1,520 (86.76 %)	
Borderline	86 (1.58%)	31 (2.06%)	26 (1.22%)	29 (1.47%)	
**CVD, %**					< 0.001
Yes	4,712 (88.71%)	1,538 (86.01%)	1,575 (88.45%)	1,599 (91.67%)	
No	564 (11.29%)	229 (13.99%)	191 (11.55%)	144 (8.33%)	
**Mortality, %**					< 0.001
Yes	4,551 (86.04%)	1,480 (83.17%)	1,515 (85.57%)	1,556 (89.37%)	
No	725 (13.96%)	287 (16.83%)	251 (14.43%)	187 (10.63%)	
**Glucose (mg/dL)**	107.85 ± 23.52	109.01 ± 25.78	107.23 ± 22.22	107.30 ± 22.36	0.039
**Total cholesterol (mg/dL)**	195.05 ± 41.71	196.58 ± 42.80	195.59 ± 42.11	192.98 ± 40.10	0.030
**Triglycerides (mg/dL)**	131.24 ± 101.58	132.80 ± 105.21	130.59 ± 121.78	130.33 ± 71.14	0.730
**HDL-C (mg/dL)**	53.53 ± 15.79	54.00 ± 15.17	53.52 ± 15.93	53.08 ± 16.25	0.264
**LDL-C (mg/dL)**	114.02 ± 24.16	113.62 ± 24.60	115.31 ± 5.04	113.14 ± 22.76	0.316
**Energy intake (kcal)**	77.24 ± 32.72	78.76 ± 33.76	77.19 ± 30.80	75.78 ± 33.55	0.818
**Protein intake (gm)**	77.34 ± 29.86	77.52 ± 29.87	77.89 ± 28.58	76.63 ± 31.10	0.461
**Total fat intake (gm)**	2,021.28 ± 715.02	2,044.84 ± 740.24	2,024.23 ± 672.03	1,994.91 ± 731.44	0.944
**DII**	1.29 ± 1.51	1.38 ± 1.53	1.30 ± 1.53	1.20 ± 1.58	0.002
**HEI2020**	55.24 ± 10.76	55.21 ± 10.98	55.17 ± 10.94	55.34 ± 10.35	0.887
**SII**	613.38 ± 489.52	628.09 ± 560.63	613.22 ± 445.02	598.82 ± 454.28	0.208
**CDAI**	1.88 ± 3.90	±1.88 ± 1.20	1.17 ± 0.85	6.31 ± 3.22	< 0.001

^a^Survey data were presented as weighted means ± standard errors for continuous variables, and unweighted frequencies (weighted proportions) for categorical variables. CDAI, composite dietary antioxidant index; BMI, body mass index; CVD, cardiovascular disease; HDL-C, high density lipoprotein cholesterol; PIR, poverty-to-income ratio; DII, dietary inflammation index; HEI2020, health eating index 2020; SII, systemic immune-inflammation index.

### 3.2 Association between CDAI and CVD

The multivariable logistic regression model presented in Table 2 consistently demonstrates that higher CDAI values are associated with a reduced odd of CVD. Specifically, for each unit increase in the CDAI, the likelihood of CVD decreases by approximately 6% (OR: 0.94, 95% CI: 0.92 to 0.97, *P* < 0.0001). In the fully adjusted model, when comparing the highest tertile to the lowest tertile, each unit increase in CDAI within the highest tertile is associated with a 22% reduction in CVD risk (OR: 0.78, 95% CI: 0.69 to 0.88, *P* < 0.0001). The *p*-values for the trend tests were all less than 0.0001.

**TABLE 2:** Weighted multivariate logistics regression model analysis reveals the association between CDAI and the odds of cardiovascular disease (NHANES, 1999–2018).

CVD	Model 1		Model 2		Model 3	
	**OR (95% CI)**	***p*-value**	**OR (95% CI)**	***p*-value**	**OR (95% CI)**	***p*-value**
CDAI	0.94 (0.92, 0.97)	< 0.0001	0.95 (0.92, 0.97)	< 0.0001	0.94 (0.92, 0.97)	< 0.0001
Low	1.0		1.0		1.0	
Middle	0.83 (0.67, 1.10)	0.0688	0.83 (0.68, 1.02)	0.0842	0.84 (0.67, 1.05)	0.1270
High	0.61 (0.49, 0.76)	< 0.0001	0.61 (0.49, 0.76)	< 0.0001	0.61 (0.47, 0.77)	< 0.0001
*p* for trend	0.78 (0.70, 0.87)	< 0.0001	0.78 (0.70, 0.88)	< 0.0001	0.78 (0.69, 0.88)	< 0.0001

Model 1: adjusted for none. Model 2: adjusted for age, gender, race, education level. Model 3: adjusted for age, gender, race, education level, marital status, poverty to income ratio, BMI, smoking, drinking alcohol, diabetes, energy intake, total fat intake, protein intake, HDL-c, LDL, glucose, total cholesterol, triglycerides, DII, HEI-2020, SII. CDAI tertiles: Low (−6.52 to −0.20), Middle (−0.20 to 2.75), and High (2.75 to 19.97). CI, confidence interval; OR, odds ratio; EO, ethylene oxide; BMI, body mass index; PIR, poverty-to-income ratio; CVD, cardiovascular disease; HDL-C, high density lipoprotein cholesterol; LDL, low density lipoprotein cholesterol, DII, dietary inflammation index; HEI-2020, health eating index 2020; SII, systemic immune-inflammation index.

Additionally, we investigated the associations between individual components of the CDAI (Supplementary Table 1) as well as the HEI2020, DII and SII (Supplementary Table 2) with CVD odds. In all models, vitamin E and selenium were consistently negatively correlated with the likelihood of CVD. In the fully adjusted model, vitamin E was found to reduce disease odds by 26% (OR: 0.74, 95% CI: 0.68 to 0.81), while selenium was associated with a 29% reduction (OR: 0.71, 95% CI: 0.65 to 0.77). Vitamin C also demonstrated an inverse association, leading to a 7% reduction in disease odds in model 3 (OR: 0.93, 95% CI: 0.86 to 0.98). However, no statistically significant associations were observed between HEI2020, DII or SII scores and CVD odds in any of the analytical models.

### 3.3 Association between CDAI and mortality

In the Cox regression analysis presented in Table 3, when CDAI was examined as a continuous variable, it was found to be independently associated with mortality in the fully adjusted model (HR = 0.94, 95% CI: 0.91 to 0.96), indicating a 6% reduced risk of mortality per unit increase in CDAI. When stratified by tertiles of CDAI, compared to the reference group (Low), the high CDAI group exhibited stronger associations, with HRs of 0.60 (95% CI, 0.48 to 0.75) in Model 1, 0.59 (95% CI, 0.47 to 0.74) in Model 2, and 0.55 (95% CI, 0.43 to 0.70) in Model 3, all with *p*-values less than 0.0001. The *p*-value for trend across the tertiles was also significant in all models.

**TABLE 3:** The Cox regression analysis shows the association between the CDAI with the mortality (NHANES, 1999–2018).

	Model 1		Model 2		Model 3	
	**HR (95% CI)**	***p*-value**	**HR (95% CI)**	***p*-value**	**HR (95% CI)**	***p*-value**
CDAI	0.95 (0.93, 0.97)	< 0.0001	0.95 (0.93, 0.97)	< 0.0001	0.95 (0.93, 0.97)	< 0.0001
Low	1.0		1.0		1.0	
Middle	0.85 (0.71, 1.00)	0.0620	0.84 (0.71, 1.00)	0.0607	0.82 (0.69, 0.98)	0.0321
High	0.63 (0.53, 0.76)	< 0.0001	0.64 (0.53, 0.77)	< 0.0001	0.61 (0.50, 0.74)	< 0.0001
*p* for trend	0.80 (0.73, 0.87)	< 0.0001	0.80 (0.73, 0.88)	< 0.0001	0.78 (0.71, 0.86)	< 0.0001

Model 1: adjusted for none. Model 2: adjusted for age, gender, race, education level. Model 3: adjusted for age, gender, race, education level, marital status, poverty to income ratio, BMI, smoking, drinking alcohol, diabetes, energy intake, total fat intake, protein intake, HDL-c, LDL, Glucose, Total cholesterol, Triglycerides, DII, HEI-2020, SII. CDAI tertiles: Low (−6.52 to −0.20), Middle (−0.20 to 2.75), and High (2.75 to 19.97). CI, confidence interval; OR, odds ratio; EO, ethylene oxide; BMI, body mass index; PIR, poverty-to-income ratio; CVD, cardiovascular disease; HDL-C, high density lipoprotein cholesterol; LDL, low density lipoprotein cholesterol.

We further investigated the associations of individual antioxidant components (Supplementary Table 3) along with the HEI2020, DII and SII (Supplementary Table 4) with mortality risk. Vitamin E and selenium are significantly associated with reduced mortality, with an HR of 0.82 (95% CI, 0.75 to 0.90) for vitamin E and 0.72 (95% CI, 0.65 to 0.80) for selenium in the fully adjusted model. Vitamin C shows a trend toward reduced risk in some models, while the associations for carotene and zinc are less consistent. Notably, no significant correlations were observed between mortality risk and HEI2020, DII, or SII scores in the study population.

**FIGURE 2:**
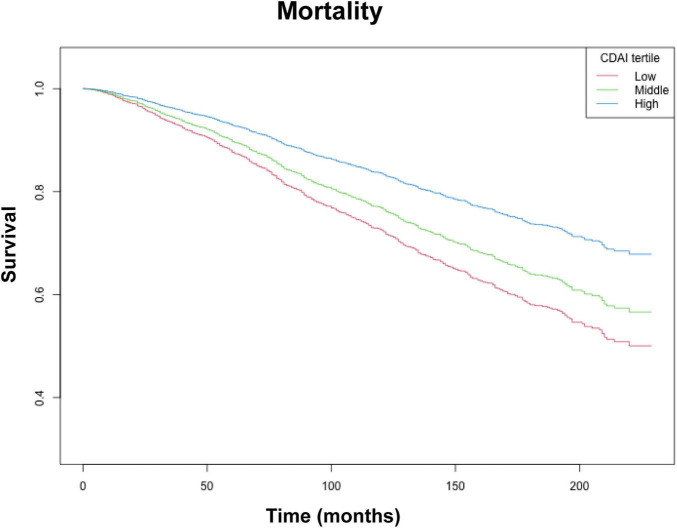
Kaplan–Meier survival analysis (NHANES, 1999–2018). CDAI tertiles: Low (−6.52 to −0.20), Middle (−0.20 to 2.75), and High (2.75 to 19.97). Adjusted for: age, race, education level, marital status, poverty to income ratio, BMI, smoking, drinking alcohol, diabetes, energy intake, total fat intake, protein intake, HDL-c, LDL, Glucose, Total cholesterol, Triglycerides, DII, HEI-2020, SII.

Kaplan–Meier survival curves of the CDAI and individual antioxidants for long-term mortality are plotted in Figure 2 and Supplementary Figure 1 In the order of hypertension people, the mortality rates decreased with increasing tertiles of the CDAI, Vitamin E, Vitamin C, Zinc, and Carotene (all log-rank *p* < 0.05).

### 3.4 Subgroup analysis

**FIGURE 3:**
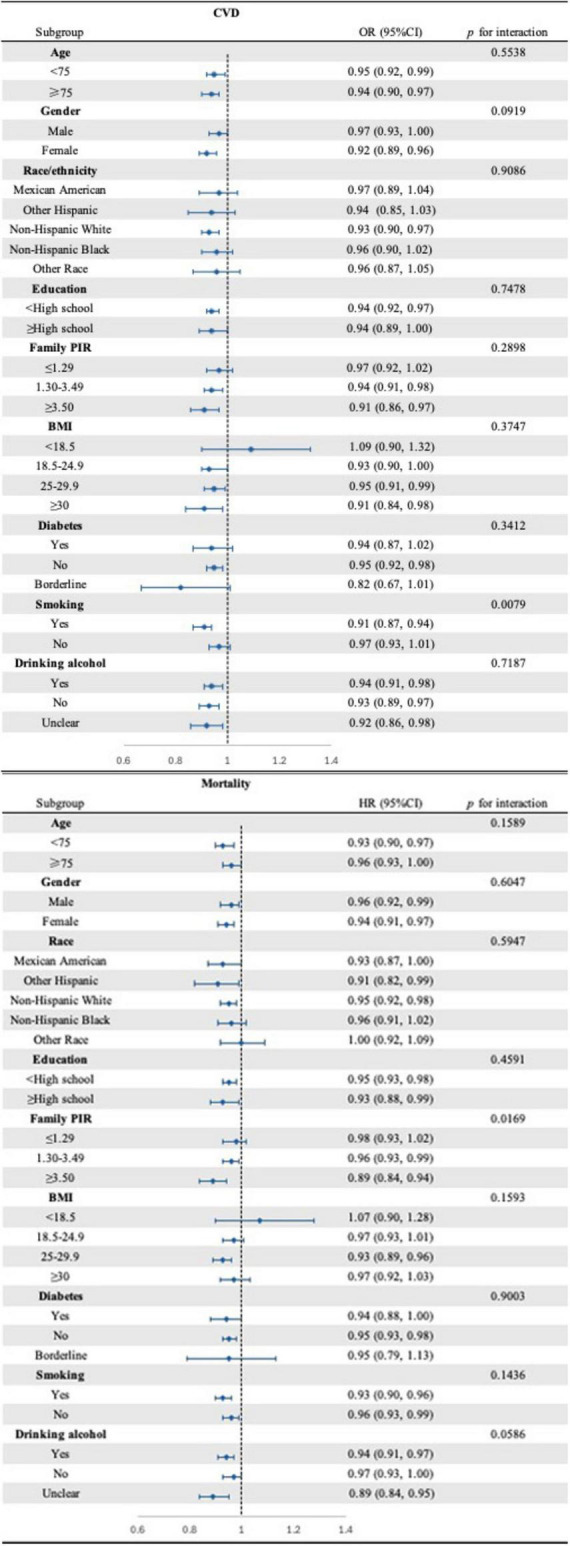
Forest plot of subgroups analysis for the associations between CDAI and CVD (top) as well as mortality (bottom) (NHANES, 1999–2018). Adjusted for: age, gender, race, education level, marital status, poverty to income ratio, BMI, smoking, drinking alcohol, diabetes, energy intake, total fat intake, protein intake, HDL-c, LDL, Glucose, Total cholesterol, Triglycerides, DII, HEI-2020, SII.

To assess the correlation between CDAI and cardiovascular disease (CVD) as well as mortality, subgroup analyses were conducted (Figure 3). Notably, most of these analyses did not reveal significant differences across the subgroups. However, a more pronounced positive association was observed between CDAI and CVD among individuals with a history of never smoking or having smoked. Similarly, the positive correlation between CDAI and mortality was more pronounced among participants with varying PIR.

### 3.5 Mediation analysis

**FIGURE 4:**
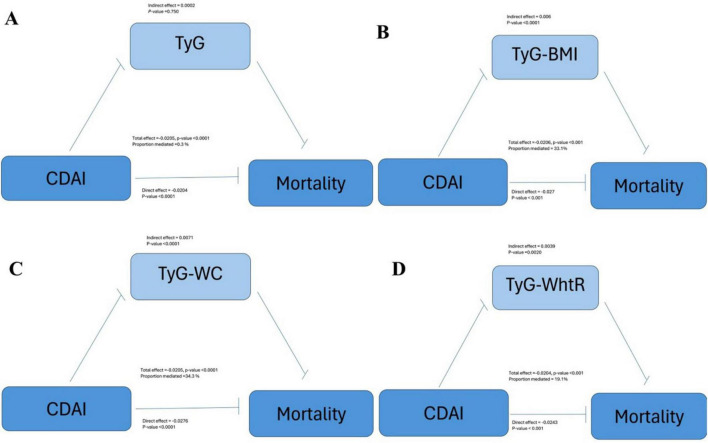
Mediating effects of **(A)** TyG, **(B)** TyG-BMI, **(C)** TyG-WC, and **(D)** TyG-WhtR on the association between CDAI and mortality (NHANES, 1999–2018). Adjusted for: age, gender, race, education level, marital status, poverty to income ratio, smoking, drinking alcohol, diabetes, energy intake, total fat intake, protein intake, HDL-c, LDL-c, DII, HEI-2020, SII.

Figure 4 illustrates that the TyG-BMI index accounts for 33.1% of the correlation between CDAI scores and mortality rates. In the examination of TyG and its related indices—TyG-WC and TyG-WHtR—their respective contributions to the mediation effect were found to be 0.3%, 34.3% and 19.1%.

We similarly examined the mediating effects of inflammatory factors (Supplementary Figure 2) such as lymphocytes, neutrophils, and immunoinflammatory factors on the relationship between CDAI and mortality and showed mediating effect values of 0.02%, 0.1%, and 0.2%, respectively.

## 4 Discussion

This study utilized both cross-sectional and longitudinal designs to examine the relationship between the CDAI and CVD in older individuals with hypertension in the United States. The findings revealed a negative association between CDAI and CVD, irrespective of whether CDAI was treated as a categorical or continuous variable. Additionally, the cohort analysis demonstrated that elevated CDAI levels were associated with a reduced risk of mortality in this population. Further mediation analyses indicated that the TyG index plays a significant mediating role in this observed relationship.

Our study demonstrated a significant negative correlation between CDAI and the likelihood of hypertension in the elderly population. This is consistent with previous studies. In the substantial E3N French prospective cohort, the total antioxidant capacity of the diet exhibited an inverse correlation with the risk of hypertension, with a 15% decrease in risk observed among those in the fifth compared to the first quintile (HR_*Q5*_: 0.85, 95% CI: 0.74 to 0.95) (35). Hypertension is not only considered a major risk factor for CVD but is also attributed to about one-third of all deaths worldwide (36, 37). Nutritional deficiencies, particularly suboptimal diets, have been associated with up to 45% of deaths attributable to cardiometabolic disease (38–40). Conversely, diets that are considered healthy, such as the Mediterranean diet, as well as antioxidants like vitamin E, have been demonstrated to have protective effects in reducing both cardiovascular morbidity and mortality. Consequently, guidelines have emphasized the importance of implementing healthy dietary strategies as a primary means of preventing hypertension and CVD (41). The importance of dietary antioxidants for cardiovascular health in the elderly population is further reinforced by our findings showing the correlation between CDAI and reduced odds of CVD and mortality. A meta-analysis of prospective cohort studies reviewed that high total antioxidant capacity (TAC) was associated with decreased risk of death from all-cause, cancer, and CVDs (combined effect size: 0.62, 0.81 and 0.71, respectively) (42). Kim et al. (43) found that the inverse association between dietary TAC and all-cause mortality (HR: 0.79, 95% CI: 0.71 to 0.87) and CVD mortality (HR: 0.74, 95% CI: 0.61 to 0.89).

However, the relationship between CDAI and mortality may not only be due to the direct effects of antioxidants but could also be closely related to metabolic health status. Our analysis revealed that the TyG index partially mediates the relationship between CDAI and mortality. The TyG index serves as a marker for IR, a well-established risk factor for the development of CVD and poor outcomes in individuals with CVD (44). Wang et al. (45) demonstrated a positive association between IR and the progression of CVD in individuals with prediabetes. In a cohort study conducted in Korea, with a median follow-up duration of 9.83 years, IR was found to significantly increase the risk of all-cause mortality, cardiovascular mortality, and adverse cardiovascular events in CVD patients by 87%, 133%, and 267%, respectively (46). Similarly, two cohort studies conducted in China found that in hospitalized patients with coronary heart disease (CHD) and hypertension, the TyG index was positively correlated with adverse outcomes, including all-cause mortality, over a one-year follow-up period (47). In contrast, Hou et al. (48) did not observe a significant link between the TyG index and mortality, which could be due to variations in the study populations and the length of follow-up. Our findings further validate this notion, showing that higher CDAI is associated with a lower risk of CVD and mortality. This highlights the potential of dietary interventions in managing cardiovascular health in older adults, particularly in preventing hypertension and related disease.

The precise mechanisms by which the CDAI influences hypertension and mortality are not yet fully elucidated. However, oxidative stress is proposed to play a central role in mediating these effects. Oxidative stress arises when there is a disproportion between antioxidants and pro-oxidants, typically marked by an accumulation of reactive oxygen species (ROS) (49). These ROS contribute to the pathogenesis of hypertension through mechanisms such as endothelial injury, vascular dysfunction, remodeling, and the activation of the sympathetic nervous system (50). Additionally, ROS are implicated in the development of insulin resistance and lipid peroxidation, which disrupt blood pressure regulation as well as glucose and lipid metabolism, thereby increasing the risk of CVD (51–53). Moreover, oxidative stress-induced reductions in nitric oxide levels further exacerbate both insulin resistance and endothelial dysfunction, which in turn heightens the risk of hypertension and diabetes (54, 55). Our mediation analysis suggests that the CDAI’s impact on reducing mortality risk may be partially mediated by a reduction in the TyG index, offering insight into potential mechanistic pathways. Reactive oxygen and nitrogen species (RONS) disrupt insulin signaling pathways (56). A transient burst of hydrogen peroxide (HO) during insulin release enhances insulin signaling by inhibiting tyrosine phosphatase activity, thus increasing basal tyrosine phosphorylation in the insulin receptor and downstream targets (57). Additionally, lipid peroxidation mediated by ROS such as hydroxyl and hydroperoxyl radicals intensifies insulin resistance (58). Impaired insulin signaling in vascular tissues can lead to endothelial dysfunction, hypertension, and atherosclerosis. The interaction between oxidative stress and impaired insulin metabolic signaling is likely central to the pathogenesis of hypertension, cardiometabolic syndrome (CMS), and CVD (59). Moreover, it is well-established that insulin resistance is associated with a decline in endogenous antioxidant defenses (60). The intricate interplay between hypertension, insulin resistance, and cardiovascular disease warrants further investigation to fully elucidate these complex relationships.

Emerging evidence suggests that genetic variations in antioxidant enzyme genes, such as glutathione S-transferases (GSTs), may modulate the effects of dietary antioxidants on cardiovascular risk (61). For instance, individuals with certain GST polymorphisms may exhibit differential responses to antioxidant-rich diets, highlighting the importance of personalized nutrition strategies (62). Additionally, other dietary components, such as fiber and polyphenols, may synergistically enhance the cardiovascular benefits of antioxidants. Fiber has been shown to improve lipid profiles and reduce systemic inflammation, while polyphenols exert anti-inflammatory and antioxidant effects that complement those of traditional antioxidants (12, 63). Beyond the TyG index, other potential mediators, such as the SII, which integrates neutrophil, lymphocyte, and platelet counts, may further elucidate the relationship between CDAI and cardiovascular outcomes (30). Elevated SII levels have been associated with increased CVD risk, suggesting that dietary antioxidants may also exert their effects through modulation of systemic inflammation (64). However, our findings do not reflect a significant mediating effect of SII, neutrophils and lymphocytes. From a public health perspective, promoting diets rich in antioxidants could be an effective strategy to reduce CVD risk and mortality in elderly hypertensive populations. Incorporating CDAI into dietary guidelines may provide a practical tool for assessing and improving dietary quality in this vulnerable group.

Future research should focus on randomized controlled trials to evaluate the effects of antioxidant supplementation on CVD outcomes. Additionally, studies exploring gene-diet interactions and personalized dietary interventions based on genetic and metabolic profiles are needed to optimize the benefits of antioxidant-rich diets (65). Comparing CDAI with other dietary indices, such as the DII and HEI, could provide a more comprehensive understanding of the role of diet in cardiovascular health (31). Furthermore, investigating the potential interactions between antioxidants and other nutrients or medications is crucial, as these factors may influence the bioavailability and efficacy of dietary antioxidants (66).

Although the results of this study provide valuable insights, there are several limitations. First, the study employed a cross-sectional design, which prevents establishing causal relationships between CDAI and hypertension, CVD, or mortality. Future prospective studies are required to confirm these associations and investigate the long-term impacts of dietary antioxidants. Second, CDAI is a composite measure, and while it reflects overall antioxidant intake, the specific biological effects may vary among individuals. Therefore, future studies should consider the impact of specific types and doses of antioxidants on hypertension and CVD risk. Third, we acknowledge that other dietary components, such as fiber and polyphenols, may also play significant roles in reducing CVD risk. These components were not explicitly included in the CDAI calculation, and their potential synergistic effects with antioxidants warrant further investigation. Fourth, the use of self-reported CVD diagnoses may introduce recall bias, which could affect the accuracy of our findings. Future studies should incorporate biomarker-based assessments (e.g., inflammatory markers, imaging data) to validate CVD outcomes and reduce potential bias. Lastly, although the TyG index played a mediating role in this study, its precise mechanisms remain unclear and warrant further investigation through mechanistic studies and clinical trials to elucidate its role in the prevention of CVD through dietary antioxidants.

## 5 Conclusion

In this nationally representative cohort study of older adults with hypertension, we observed that higher CDAI scores were significantly associated with reduced odds of CVD and all-cause mortality. These associations remained robust after adjusting for a wide range of potential confounders, including sociodemographic, lifestyle, and clinical factors. Furthermore, our mediation analysis revealed that the TyG index and its related indices partially mediated the relationship between CDAI and mortality, suggesting that the beneficial effects of dietary antioxidants may be partly explained by improvements in insulin resistance and metabolic health.

These findings underscore the potential importance of dietary antioxidants in promoting cardiovascular health and reducing mortality risk among elderly hypertensive individuals. However, it is important to note that this study is observational in nature, and thus, the observed associations do not imply causation. Future research, particularly randomized controlled trials, is needed to confirm these findings and to explore the specific mechanisms by which dietary antioxidants influence cardiovascular outcomes. Additionally, further investigation into the role of other dietary components, such as fiber and polyphenols, as well as genetic factors, may provide a more comprehensive understanding of the interplay between diet, metabolic health, and cardiovascular risk.

In conclusion, our study highlights the potential benefits of a diet rich in antioxidants for reducing CVD risk and mortality in older adults with hypertension. These findings support the incorporation of dietary antioxidant indices, such as CDAI, into public health strategies aimed at improving cardiovascular health in this vulnerable population.

## Data Availability

The datasets presented in this study can be found in online repositories. The names of the repository/repositories and accession number(s) can be found below: The survey data are publicly available on the internet for data users and researchers throughout the world (www.cdc.gov/nchs/nhanes/).

## Footnotes

1
https://wwwn.cdc.gov/nchs/nhanes/tutorials/default.aspx
