# Supplementary material for: Mediating role of triglyceride glucose-related index in the associations of composite dietary antioxidant index with cardiovascular disease and mortality in older adults with hypertension: a national cohort study
Source: Front Nutr. 2025 Apr 23;12:1574876. doi: 10.3389/fnut.2025.1574876 (PMC12055501; doi:10.3389/fnut.2025.1574876)
Supplement: Supplementary file 1 [file Table_1.docx]

**Supplementary Materials**

**事后样本检验**

**Table S1. Multivariate logistic regression model analyses reveal the relationship between the components of CDAI and the odds of cardiovascular disease**

| **CVD** | **Model 1** | | **Model 2** | | **Model 3** | |
| --- | --- | --- | --- | --- | --- | --- |
|  | OR (95% CI) | *p* -value | OR (95% CI) | *p -*value | OR (95% CI) | *p* -value |
| **Vitamin A** | 0.93 (0.84, 1.04) | 0.1928 | 0.94 (0.85, 1.04) | 0.2521 | 0.96 (0.85, 1.07) | 0.4456 |
| **Vitamin E** | 0.83 (0.75, 0.91) | <0.0001 | 0.83 (0.76, 0.91) | 0.0001 | 0.83 (0.75, 0.92) | 0.0003 |
| **Vitamin C** | 0.91 (0.83, 1.00) | 0.0461 | 0.91 (0.83, 1.00) | 0.0544 | 0.99 (0.91, 1.09) | 0.9072 |
| **Carotene** | 0.93 (0.85, 1.01) | 0.0841 | 0.94 (0.86, 1.02) | 0.1368 | 0.95 (0.85, 1.07) | 0.4207 |
| **Selenium** | 0.76 (0.69, 0.84) | <0.0001 | 0.77 (0.69, 0.85) | <0.0001 | 0.74 (0.67, 0.83) | <0.0001 |
| **Zinc** | 0.86 (0.77, 0.96) | 0.0055 | 0.86 (0.77, 0.96) | 0.0053 | 0.85 (0.76, 0.95) | 0.0034 |

Model 1: adjusted for none.
Model 2: adjusted for age, gender, race, education level.

Model 3: adjusted for adjusted for age, gender, race, education level, marital status, poverty to income ratio, BMI, smoking, drinking alcohol, diabetes, energy intake, total fat intake, protein intake, HDL-c, LDL, Glucose, Total cholesterol, Triglycerides, DII, HEI-2020, SII.

Abbreviations: CI, confidence interval; OR, odds ratio; EO, ethylene oxide; BMI, body mass index; PIR, poverty-to-income ratio; CVD, cardiovascular disease; HDL-C, high density lipoprotein cholesterol; LDL, low density lipoprotein cholesterol; DII, dietary inflammation index; HEI-2020, health eating index 2020; SII, systemic immune-inflammation index;

**Table S2. Multivariate logistic regression model analyses reveal the relationship between HEI2020, DII, SII and the odds of cardiovascular disease**

| **CVD** | **Model 1** | | **Model 2** | | **Model 3** | |
| --- | --- | --- | --- | --- | --- | --- |
|  | OR (95% CI) | *p* -value | OR (95% CI) | *p -*value | OR (95% CI) | *p* -value |
| **HEI2020** | 1.00 (1.00, 1.01) | 0.3663 | 1.00 (0.99, 1.01) | 0.5345 | 1.00 (0.99, 1.01) | 0.7115 |
| **DII** | 1.00 (0.95, 1.06) | 0.8929 | 1.00 (0.94, 1.06) | 0.9740 | 1.01 (0.93, 1.08) | 0.8883 |
| **SII** | 1.00 (1.00, 1.00) | 0.3993 | 1.00 (1.00, 1.00) | 0.4516 | 1.00 (1.00, 1.00) | 0.7739 |

Model 1: adjusted for none.
Model 2: adjusted for age, gender, race, education level.

Model 3: adjusted for adjusted for age, gender, race, education level, marital status, poverty to income ratio, BMI, smoking, drinking alcohol, diabetes, energy intake, total fat intake, protein intake, HDL-c, LDL, Glucose, Total cholesterol, Triglycerides, CDAI.

Abbreviations: CI, confidence interval; OR, odds ratio; EO, ethylene oxide; BMI, body mass index; PIR, poverty-to-income ratio; CVD, cardiovascular disease; HDL-C, high density lipoprotein cholesterol; LDL, low density lipoprotein cholesterol; DII, dietary inflammation index; HEI-2020, health eating index 2020; SII, systemic immune-inflammation index;

Table S3. The Cox regression analysis shows the association between the components of CDAI with the mortality.

|  | Model 1 | | Model 2 | | Model 3 | |
| --- | --- | --- | --- | --- | --- | --- |
|  | HR (95%CI) | *p* -value | HR (95%CI) | *p* -value | HR (95%CI) | *p* -value |
| **Vitamin A** | 1.08 (1.00, 1.17) | 0.0463 | 1.09 (1.00, 1.17) | 0.0377 | 1.10 (1.01, 1.20) | 0.0226 |
| **Vitamin E** | 0.77 (0.71, 0.84) | <0.0001 | 0.77 (0.71, 0.84) | <0.0001 | 0.76 (0.70, 0.84) | <0.0001 |
| **Vitamin C** | 0.98 (0.91, 1.06) | 0.5634 | 0.98 (0.91, 1.06) | 0.6042 | 1.03 (0.95, 1.11) | 0.5009 |
| **Carotene** | 1.03 (0.97, 1.09) | 0.3559 | 1.04 (0.97, 1.10) | 0.0399 | 1.02 (0.95, 1.11) | 0.5554 |
| **Selenium** | 0.73 (0.67, 0.79) | <0.0001 | 0.73 (0.67, 0.79) | <0.0001 | 0.72 (0.66, 0.79) | <0.0001 |
| **Zinc** | 0.91 (0.83, 0.99) | 0.0346 | 0.91 (0.83, 0.99) | 0.0355 | 0.91 (0.82, 0.99) | 0.0382 |

Model 1: adjusted for none.
Model 2: adjusted for age, gender, race, education level.

Model 3: adjusted for adjusted for age, gender, race, education level, marital status, poverty to income ratio, BMI, smoking, drinking alcohol, diabetes, energy intake, total fat intake, protein intake, HDL-c, LDL, Glucose, Total cholesterol, Triglycerides, DII, HEI-2020, SII.

Abbreviations: CI, confidence interval; OR, odds ratio; EO, ethylene oxide; BMI, body mass index; PIR, poverty-to-income ratio; CVD, cardiovascular disease; HDL-C, high density lipoprotein cholesterol; LDL, low density lipoprotein cholesterol; DII, dietary inflammation index; HEI-2020, health eating index 2020; SII, systemic immune-inflammation index;

Table S4. The Cox regression analysis shows the association between HEI2020, SII, DII with the mortality.

|  | Model 1 | | Model 2 | | Model 3 | |
| --- | --- | --- | --- | --- | --- | --- |
|  | HR (95%CI) | *p* -value | HR (95%CI) | *p* -value | HR (95%CI) | *p* -value |
| **HEI2020** | 1.00 (1.00, 1.101 | 0.2235 | 1.00 (1.00, 1.01) | 0.1923 | 1.01 (1.00, 1.02) | 0.0676 |
| **DII** | 1.03 (0.98, 1.08) | 0.2676 | 1.02 (0.97, 1.08) | 0.3438 | 1.05 (0.99, 1.11) | 0.1138 |
| **SII** | 1.00 (1.00, 1.00) | 0.8779 | 1.00 (1.00, 1.00) | 0.9936 | 1.00 (1.00, 1.00) | 0.7335 |

Model 1: adjusted for none.
Model 2: adjusted for age, gender, race, education level.

Model 3: adjusted for adjusted for age, gender, race, education level, marital status, poverty to income ratio, BMI, smoking, drinking alcohol, diabetes, energy intake, total fat intake, protein intake, HDL-c, LDL, Glucose, Total cholesterol, Triglycerides, CDAI.

Abbreviations: CI, confidence interval; OR, odds ratio; EO, ethylene oxide; BMI, body mass index; PIR, poverty-to-income ratio; CVD, cardiovascular disease; HDL-C, high density lipoprotein cholesterol; LDL, low density lipoprotein cholesterol; DII, dietary inflammation index; HEI-2020, health eating index 2020; SII, systemic immune-inflammation index;


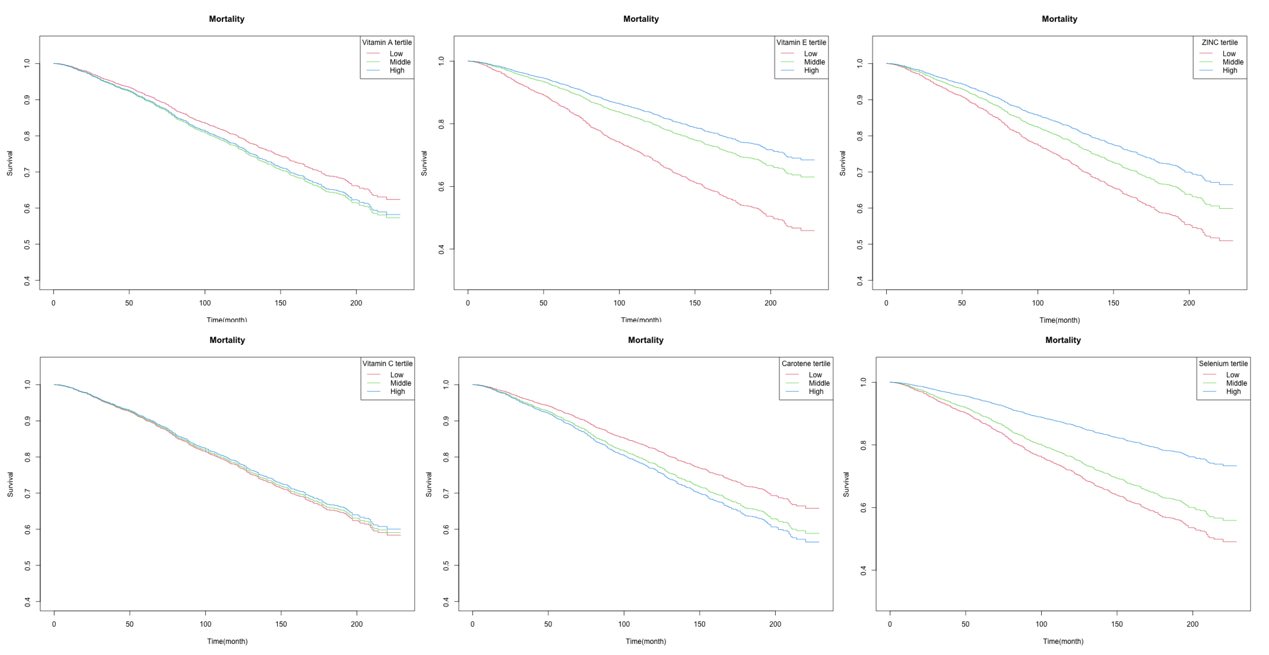


**Fig. S1. Kaplan-Meier survival analysis**

**
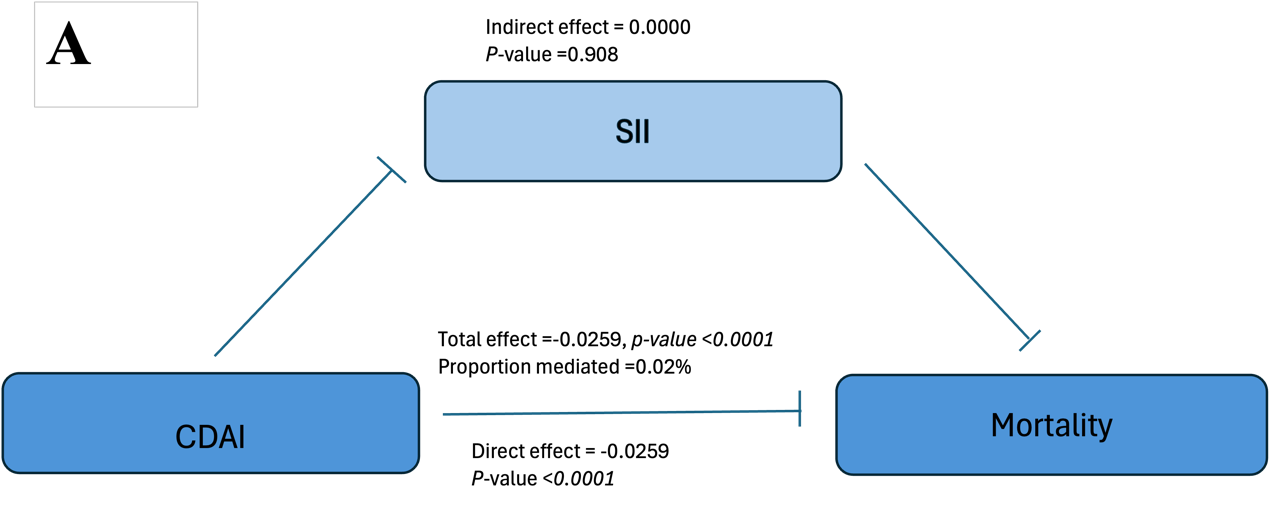
**

**
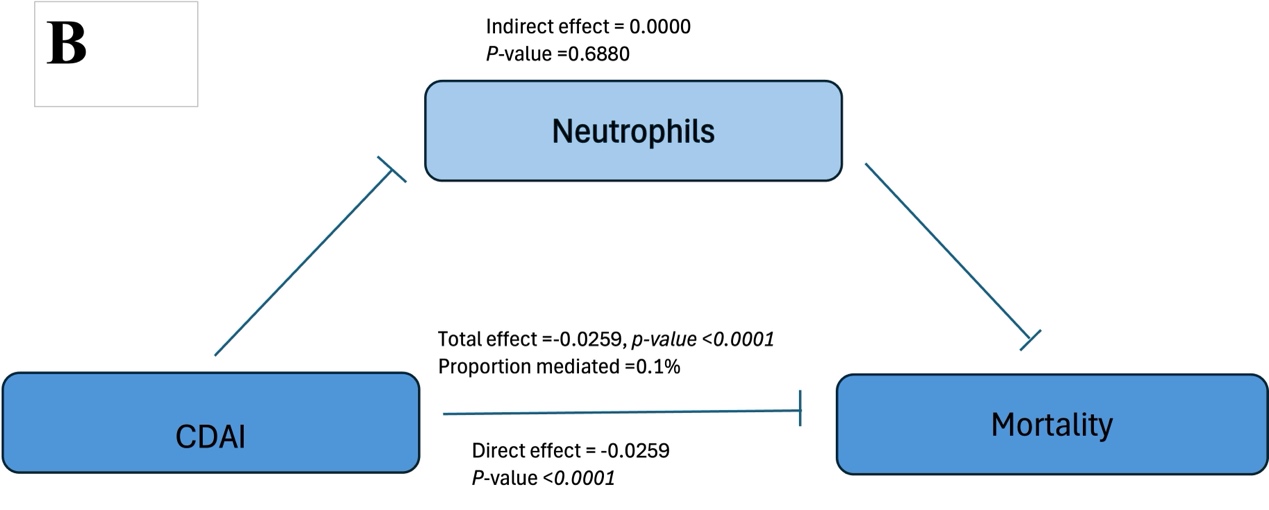
**

**
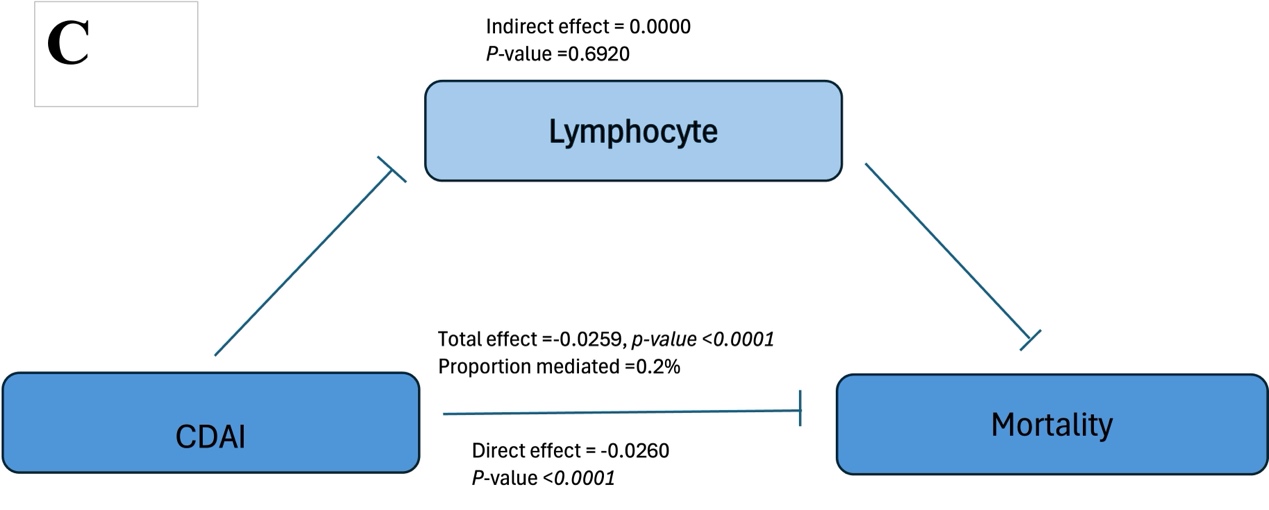
**

Fig. S2. Mediation effect of inflammatory factor for the association between CDAI and mortality (A: SII; B: Neutrophils; C: Lymphocyte)
